# Identifying stress-relevant events in everyday nursing practice using psychophysiological outcomes - an observational multimethod study

**DOI:** 10.1186/s12912-026-05434-w

**Published:** 2026-09-29

**Authors:** Elizabeth A. M. Michels, Christian Rominger, Andreas R. Schwerdtfeger, Georg F. Kurze, Justus Klein, Jonas L. Steinhäuser-Meerz, Hannah Tolle, Magdalena K. Wekenborg

**Affiliations:** 1 Else Kröner Fresenius Center (EKFZ) for Digital Health, Faculty of Medicine Carl Gustav Carus, TUD Dresden University of Technology, Fetscherstraße 74, 01307 Dresden, Germany; 2 Health Psychology, University of Graz, Graz, Austria; 3 Department of Medicine I, Gastroenterology and Hepatology, University Hospital Carl Gustav Carus, Dresden, Germany

**Keywords:** Nursing, Occupational stress, Ecological momentary assessment, Heart rate variability, Wearable sensors, Multilevel modeling, Job demands-resources, Clinical work events

## Abstract

**Background:**

Stressful working conditions contribute substantially to nurses’ sickness absence and workforce shortages. However, prior research has largely focused on general job demands and resources, providing limited insight into which everyday work events are associated with acute stress responses. This study examined event-related observer-rated and physiological stress indicators during nurses’ work using ecological momentary assessment combined with ambulatory heart rate variability (HRV) monitoring.

**Methods:**

A non-interventional, multimethod field study was conducted with nurses from a German university hospital. During an 8-hour shift, trained observers continuously recorded work events and rated momentary stress, while HRV (root mean square of successive differences; lnRMSSD) was assessed via wearable electrocardiography. Events were categorized into clinical care burden, organizational burden, and positive events. Associations between event categories and observer-rated stress and lnRMSSD, controlling for physical activity, were examined using multilevel models. Additional analyses examined individual characteristics (age, sex, chronic stress) and patient call system events, including call reasons.

**Results:**

Data from up to 85 nurses and 1,617 event-level observations were analyzed. Clinical care burden was consistently associated with higher observer-rated stress compared to other event categories, while positive events were associated with the lowest observer-rated stress. For HRV, clinical care burden was associated with lower lnRMSSD than organizational burden, whereas no significant differences were observed between positive events and other categories. An exploratory association between sex and lnRMSSD was observed, with male nurses showing higher values. Chronic stress was associated with higher observer-rated stress. Patient call system events did not differ significantly from non-call work events in observer-rated stress or HRV. However, call reason was associated with observer-rated stress, with calls related to comfort and service issues rated as less stressful than acute medical needs and care and mobilization support calls. No significant HRV associations were found.

**Conclusions:**

Psychophysiological indicators in nursing vary across everyday work events, with clinical care burden emerging as the most consistently stress-associated event category. The combination of event-based assessment and ambulatory physiological monitoring is feasible in routine clinical practice and enables identification of stress-relevant situations with high ecological validity. These findings may inform targeted interventions to improve nurses’ working conditions.

**Supplementary Information:**

The online version contains supplementary material available at https://doi.org/10.1186/s12912-026-05434-w.

## Introduction

The global demand for nurses continues to rise due to aging populations and growing healthcare needs. Yet this trend stands in sharp contrast to an already existing global shortage estimated at 4.5 million nurses by 2030 [1]. Major contributors to this current shortage are high rates of sickness absence and nurses’ decision to leave the profession, both of which are linked to stressful working conditions [2–4]. Improving working conditions is therefore essential, not only to protect nurses’ well-being but also to strengthen workforce stability and ensure the quality and stability of patient care. As a first step toward this goal, identifying stress-relevant events in everyday clinical work seems crucial for developing targeted changes in clinical practice. To do so, it is necessary to clarify how stress is conceptualized in the present context.

**Fig. 1:**
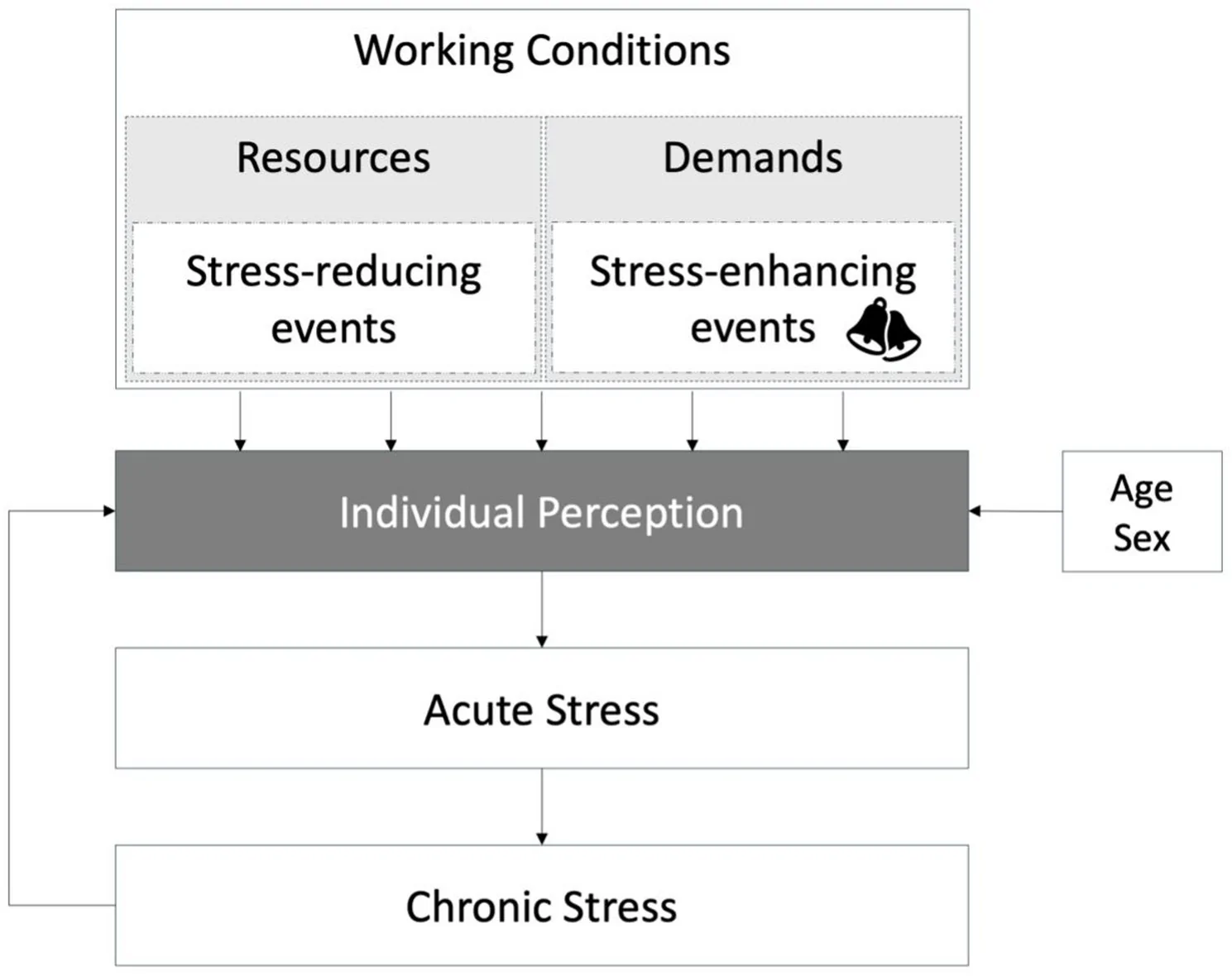
Authors’ conceptual integration of the job demands-resources and transactional stress models. Note. This figure represents the authors’ own conceptual integration of the Job Demands–Resources model [17] and the Transactional Stress Model [5]. Working conditions consist of a multitude of stress-enhancing and stress-reducing events representing demands and resources, respectively. These events are processed through individual perception, influenced by factors such as age and sex, leading to acute and, over time, chronic stress

Stress is understood as a multidimensional construct, encompassing both psychological and physiological components [5, 6]. Psychologically, stress occurs when perceived demands (i.e., stressors) exceed coping resources and is accompanied by negative affect, such as feelings of helplessness and uncontrollability [5]. Physiologically, acute stress involves changes in the activity of central regulatory systems, including increased sympathetic activity and reduced parasympathetic activity [5, 6]. While acute stress responses can be adaptive [7, 8], sustained activation contributes to chronic stress [7, 9] and adverse health outcomes, including depression and cardiovascular disease, the leading causes of mortality worldwide [10–14]. Occupational settings, in which individuals are repeatedly exposed to demanding situations over extended periods, represent a key context in which acute stress responses may accumulate and transition into chronic stress [15]. In light of these severe consequences of chronic stress [16], theoretical models have sought to explain how working conditions (e.g., workload, interpersonal relationships) contribute to acute and chronic stress. One of the most influential frameworks is the Job Demands-Resources (JD-R) model [17]. The JD-R model distinguishes between job demands (e.g., workload, role ambiguity) and job resources (e.g., supervisor support, autonomy), proposing that high demands increase stress risk, whereas resources mitigate these effects [17]. Although the model conceptualizes demands and resources as relatively stable job characteristics, growing evidence suggests that their manifestation and effects fluctuate substantially within individuals over time [18]. Recent within-person research has demonstrated considerable variability in both work factors and employee outcomes, indicating that stress-related processes are inherently dynamic rather than static [18–20]. In line with these findings, job demands and resources can be understood as being experienced and enacted in everyday work through discrete situational events and interactions. We therefore conceptualize discrete work events as situational manifestations of broader job demands and resources. Because many individual work events may reflect similar underlying demands or resources, they can also be understood at an intermediate level of abstraction by grouping conceptually related events into broader event categories. These categories remain more closely tied to concrete everyday clinical situations than the broad job demands and resources described in the JD-R framework, thereby providing an intermediate level of analysis linking stable working conditions with momentary psychophysiological stress responses. Drawing on the Transactional Stress Model, we assume that these individual work events are cognitively appraised and may trigger psychological and physiological stress responses, reflected in observer-rated stress and cardiac autonomic regulation indexed by HRV. This event-based perspective provides a link between stable working conditions and momentary stress processes in everyday nursing practice. This conceptual perspective is illustrated in Fig. 1, which integrates the Job Demands-Resources model with the Transactional Stress Model to depict how discrete work events give rise to stress responses.

The JD-R framework has been widely applied in nursing research, consistently identifying workload, time pressure, and emotional demands as key demands, and supervisor support, autonomy, and positive interpersonal relationships as relevant resources [4, 21, 22]. This level of analysis extends beyond JD-R-based research: The broader literature on occupational stress in nursing predominantly conceptualizes working conditions in terms of general demands and resources as well [2, 3, 23–30]. While this body of research has been instrumental in establishing the link between working conditions and nurses’ stress, knowing that nurses face high workload or lack supervisory support offers limited guidance for pinpointing concrete everyday work events that could be targeted for change. A more fine-grained approach is therefore needed to identify which specific events in daily clinical practice are associated with higher or lower acute stress responses in nurses. Indeed, some studies have already moved in this direction by incorporating event-based approaches asking nurses to report specific work events. However, these assessments are typically retrospective and aggregated, focusing on event frequency rather than on situational stress levels [4, 22, 31]. For example, Sinclair et al. (2015) asked nurses to rate the frequency of positive and negative work events, which were subsequently grouped into broader categories. Although negative events were associated with adverse health outcomes, the retrospective and aggregated nature of the assessment does not allow conclusions about acute stress responses in daily clinical work.

Recent technological advances have increasingly facilitated unobtrusive psychophysiological assessments in real-world settings. A particularly suitable approach to identify stress-relevant work events is ecological momentary assessment (EMA), which involves repeated, real-time assessments of individuals’ experiences in their natural environments, thereby minimizing recall bias and maximizing ecological validity [32]. When combined with concurrent physiological measurements, EMA allows acute stress to be examined as a psychophysiological process as it unfolds during clinical work.

To date, EMA has rarely been combined with ambulatory psychophysiological measurements (e.g., electrocardiogram [ECG]) to examine acute stress in everyday nursing practice. A small number of studies have applied ambulatory cardiovascular monitoring during nurses’ work shifts, demonstrating the general feasibility of psychophysiological stress assessment in everyday nursing practice [28, 33–36]. However, these studies did not focus on identifying which specific events in nurses’ daily clinical practice are associated with changes in psychophysiological stress.

Moreover, these studies assessed physiological stress markers directly during work, whereas psychological stress was typically captured only retrospectively. This approach is understandable given the need to avoid disrupting nurses’ workflow. Given that retrospective assessments of affective states are prone to systematic recall biases and show limited agreement with momentary experiences [37, 38], this mismatch in assessment timing may obscure their interplay, highlighting the importance of concurrent real-time assessment of both psychological and physiological stress in everyday work settings.

To date, the study by Johnston et al. [39] appears to be the only one that has addressed this gap by combining real-time ambulatory ECG monitoring with ecological diary assessments to examine experienced stress during nurses’ everyday clinical work. However, their design relied on time-based sampling at fixed 90-minute intervals, which may miss stress-relevant events occurring between assessment points, particularly when events are infrequent or irregular [32, 40]. Moreover, Johnston et al. assessed global appraisals of job demands rather than documenting specific work events, leaving unanswered which concrete everyday events are associated with changes in nurses’ stress levels [39].

In contrast, the present study adopts an event-contingent assessment strategy, in which structured observations are combined with continuous ECG monitoring during nurses’ regular work shifts. Heart rate variability (HRV), derived from continuous ECG recordings, was chosen as the physiological stress indicator because it can be unobtrusively assessed during routine clinical work using wearable sensors. While its exact physiological underpinnings continue to be discussed [41, 42], HRV is widely considered to reflect cardiac autonomic regulation. Lower HRV is generally interpreted as reduced parasympathetic (vagal) activity, which has been linked to heightened physiological arousal and reduced regulatory capacity. To complement the physiological assessment, psychological stress was operationalized using observer-rated momentary stress rather than nurses’ self-reports, as repeated self-report assessments would substantially disrupt routine clinical work. Although observer ratings do not directly capture the subjective cognitive appraisal processes emphasized in the Transactional Stress Model, they provide a minimally intrusive behavioral indicator that can be synchronized with continuous physiological monitoring. Specifically, rather than relying on fixed sampling intervals, psychophysiological measurements were anchored to predefined, clinically meaningful work events. Guided by the JD-R framework [17], our approach captures both potentially stress-inducing events (i.e., demands) and positive, potentially stress-buffering events (i.e., resources).

The following main research question was examined: Which events during nurses’ everyday clinical work are associated with changes in psychological (observer-rated stress) and physiological (HRV) stress indicators? To answer this, we focused on (a) observer-rated momentary stress levels as a psychological indicator of stress and (b) heart rate variability (HRV) as a physiological indicator of stress.

As a first Sub-question, individual characteristics were examined, as empirical evidence shows that factors such as age, sex, and chronic stress modulate psychophysiological stress levels [43–45]. Sub-question 1: How do individual characteristics (i.e., age, sex, chronic stress) influence nurses’ event-related psychological (observer-rated stress) and physiological (HRV) stress indicators?

As a second Sub-question, patient call system events were examined separately, as frequent alarm-related interruptions represent a major source of stress for healthcare professionals and a risk to patient safety while their acute psychophysiological stress impact in real-world nursing practice remains largely unexplored [46]. Sub-question 2: Do patient call system events differ from non-call work events in everyday clinical practice in their impact on nurses’ psychological (observer-rated stress) and physiological (HRV) stress indicators, and does the specific reason for the call influence the stress indicators?

## Methods

### Study design

This study employed a non-interventional, multimethod field design to identify stress-relevant events directly during nurses’ regular work shifts.

### Participants

**Fig. 2:**
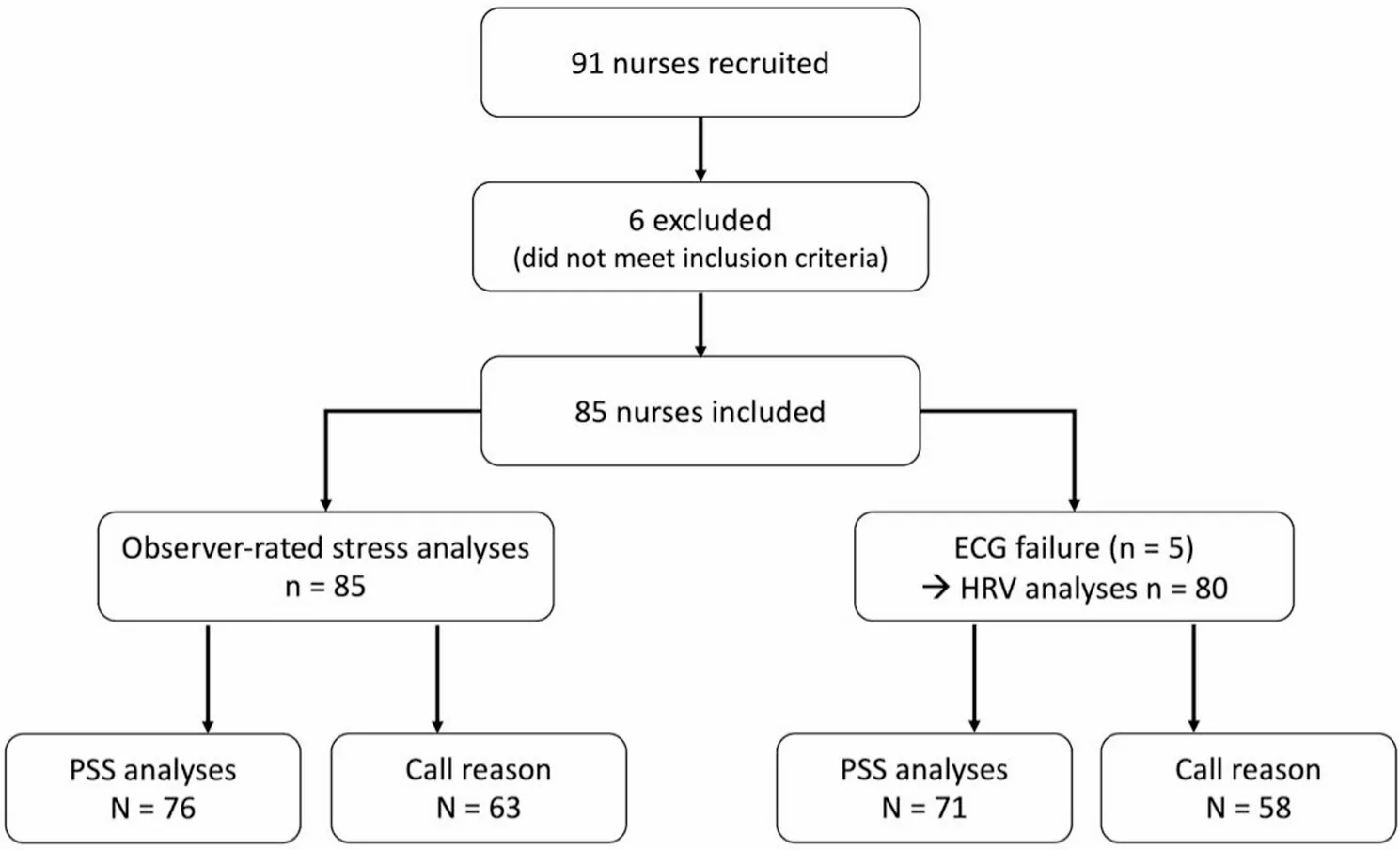
Participant flow and analytical samples. The figure summarizes participant inclusion, exclusions, and the analytical samples used for the primary analyses, chronic stress analyses, and call reason analyses

A total of 91 nurses employed at a German university hospital participated in the study between April 2024 and June 2025. Inclusion criteria were current employment in nursing (e.g., as a staff nurse or ward manager), age of at least 18 years, and sufficient proficiency in German language. Nurses worked in a variety of clinical settings, including general wards, day clinics, and intensive care units (ICUs). All nurses participated voluntarily and provided written informed consent prior to data collection. Participants received €20 compensation and individualized feedback on their HRV and selected questionnaire results. Sample size considerations were based on simulation-based reference tables for multilevel models [47] indicating that approximately 80 participants would be sufficient to detect small-to-moderate Level-1 effects given the present data structure. After data screening, 6 participants were excluded for not meeting the inclusion criteria and 5 due to technical problems with the ECG recordings, resulting in final samples of 80 nurses for the HRV analyses and 85 for the analyses of observer-rated stress. For analyses addressing Sub-question 1, which included individual characteristics (age, sex, and chronic stress), a reduced Level-2 sample was available (observer-rated stress: *n* = 76; HRV: *n* = 71) due to missing questionnaire data (PSS-10). Thus, the achieved sample size approached the target defined in the power analysis (see Table 1 for more information about the sample characteristics). Figure 2 summarizes participant flow, including the analytical samples used for the primary, chronic stress, and patient call reason analyses.

### Procedure

Data collection focused on an EMA-based psychophysiological field assessment conducted during a regular 8-hour work shift (starting at either 6:00 or 14:00), which formed the basis for the present analyses. At the beginning of each shift, nurses provided written informed consent, were fitted with an ECG sensor attached via a chest strap (EcgMove4; movisens GmbH, Karlsruhe, Germany), and a brief 2-minute resting baseline was recorded while sitting quietly. During this shift, trained observers accompanied the nurses, continuously documenting predefined potentially stress-relevant events and rating nurses’ momentary stress levels as perceived by the observer in real-time using the movisensXS application (movisens GmbH, Karlsruhe, Germany) on a tablet. Events and observer-rated stress were recorded in real time to minimize recall bias. To guide the documentation, 31 predefined events were chosen in collaboration with experienced nurses, supervisors, and occupational health psychology experts, ensuring that the events reflected both the clinical realities and psychosocial aspects of everyday nursing practice. 6 events were predefined as potentially stress-reducing (e.g., spontaneous support from colleagues, expressions of gratitude from patients), whereas the remaining events were predefined as potentially stress-enhancing (e.g., patient admission or discharge, missing equipment, searching for assistance; for a complete list of the predefined work-event types used in the primary event classification and the patient call reasons, see Supplementary Table 1). This setup enabled synchronized recording of observational and HRV data.

After the observed shift, nurses completed additional questionnaires at home, either online via LimeSurvey (LimeSurvey GmbH, Hamburg, Germany; http://www.limesurvey.org) or on paper. Of these, only measures of chronic stress, age, and sex were considered in the present analyses. Additional measures, including 24-hour ECG recordings, hair samples for steroid analysis, and sleep data, were also collected but not reported in the present paper.

### Materials

#### Self-reported measure

The Perceived Stress Scale (PSS-10; [48]) was used to measure chronic stress over the last month. The questionnaire consists of 10 items rated on a 5-point Likert scale (1 = never to 5 = often). Reliability analyses revealed good reliability in the present sample (Cronbach’s α = 0.86; McDonald’s ωt = 0.89; ωh = 0.72). 76 participants completed this questionnaire.

#### Observer-based event documentation and observer-rated stress

Throughout each shift, trained observers continuously recorded potentially stress-relevant work events and rated the nurses’ observed stress level on a visual analogue scale (VAS) ranging from 1 (not at all) to 10 (very strong). To promote standardized observations and reduce observer-related measurement bias, all observers completed a multi-stage training program consisting of theoretical instruction on the concept of stress, study procedures, predefined event categories, and a structured observer-based stress rating approach, as well as practical exercises using standardized video material to calibrate stress ratings. In addition, observers received training in recognizing behavioral indicators of stress (e.g., visible tension, hurried movements, changes in facial expression, voice, or body language) and completed a supervised pilot observation on a clinical ward before conducting independent assessments. Observers were instructed to base stress ratings on these observable behavioral indicators rather than on the objective difficulty, urgency, or presumed demands of the task itself. The predefined events were displayed within the movisensXS application interface, allowing observers to select the appropriate events for each situation directly on the tablet. Observer-rated stress was used as a behavioral indicator of momentary psychological stress. However, it should be noted that observer ratings do not directly assess nurses’ subjective cognitive appraisal of events as conceptualized in the Transactional Stress Model [5], but rather capture externally observable manifestations of stress.

During data analysis, it became evident that the frequency of event occurrences was highly uneven. Therefore, all documented events other than patient call system events were subsequently summarized into 3 overarching categories: Clinical care burden (e.g., disoriented patient requiring attention), Organizational burden (e.g., sudden tasks exceeding staff capacity), and Positive events (e.g., patient expressing verbal gratitude). The initial clustering was performed using a Large Language Model (LLM; ChatGPT-5), which was instructed to assign all events to exactly 3 higher-level thematic clusters based on their underlying cause or context, generate category names, and allocate each event to one cluster only. The resulting classification was subsequently reviewed by the study team and independently evaluated by 2 domain experts. Both experts confirmed all category assignments. The final categorization was therefore accepted without modification. The full prompt is provided in Supplementary Material S1. Patient call system events were intentionally excluded from the 3 broader event categories and analyzed separately because of their high relevance for stress and stress-related pathologies among nurses [2], as well as their considerable potential for digital innovation in clinical workflows. When a patient call system event was selected by the observer, additional information on the call was recorded. Specifically, observers documented whether the observed nurse was responsible for and responded to the call. If these criteria were met, observers selected 1 of 15 predefined call reasons (e.g., empty infusion, assistance with toileting, pain) and rated momentary observed stress. Thus, call reasons represented a subordinate classification of eligible patient call system events rather than separate work events. If the nurse was not responsible for the call and/or did not respond, only the observer-rated stress rating was completed. For the exploratory subgroup analyses of call reasons, only patient call system events with an available and classifiable call reason were included. Events with missing or non-classifiable call reasons (e.g., unclear or unlisted) were excluded from these analyses. For the binary analyses comparing patient call system events with non-call work events, patient call system events were included when the observed nurse was responsible for the call, irrespective of whether the nurse responded. The comparison group comprised all non-call work events and was not subject to this additional criterion. Accordingly, it included work events observed on wards both with and without a patient call system.

#### Interrater reliability

To assess interrater reliability, double ratings were conducted for 17 participants by pairs of independent observers. Only observations coded by both raters at approximately the same time point (± 1 min) were included. Interrater reliability of the stress ratings, assessed using the intraclass correlation coefficient (ICC [1, 3]), was 0.449 on average across participants. Agreement in event selection, assessed using Cohen’s κ, was 0.670. These agreement estimates should be considered when interpreting the findings.

#### Sensor-based physiological measures

Physiological data were collected using the EcgMove4 sensor (movisens GmbH, Karlsruhe, Germany), which continuously recorded ECG signals for deriving HRV data at a sampling rate of 1024 Hz with 12-bit resolution. The device also captured movement data through an integrated 3-dimensional accelerometer (64 Hz) for deriving Metabolic Equivalent of Task (MET) data. Physiological data were processed using *DataAnalyzer* software (movisens GmbH, Karlsruhe, Germany) with the Energy Expenditure and Cardio/HRV analysis packages, and synchronized with the EMA data using the *DataMerger* tool (movisens GmbH, Karlsruhe, Germany), resulting in a combined tabular dataset for statistical analysis. HRV preprocessing, including artifact detection, filtering of implausible RR intervals, identification of valid normal-to-normal (NN) intervals, and exclusion of invalid segments, was performed automatically by the movisens Cardio/HRV algorithms. No additional manual artifact correction or editing of RR intervals was performed. Of the 1,667 classified non-call work-event-level observations, 50 were excluded because observer-rated stress values were missing, leaving 1,617 observations for the observer-rated stress analyses. Of these 1,617 event windows, 299 (18.5%) did not yield a valid HRV estimate and were excluded, resulting in 1,318 observations for the HRV analyses.

#### Derived ambulatory HRV metrics

Within our analyses, we focused on the time-domain HRV measure root mean square of successive differences between successive RR intervals (RMSSD). RMSSD predominantly reflects parasympathetic cardiac control and can be reliably estimated from short-term ECG recordings [49]. It is therefore well suited to ambulatory designs and is less sensitive to uncontrolled breathing than frequency-domain HRV measures [50]. Furthermore, RMSSD has been frequently used as an HRV indicator in previous EMA studies [51–53], making it a suitable reference metric for the present study. In line with established standards for short-term HRV assessment recommending 5-minute recordings for reliable RMSSD estimation [49], mean HRV values were calculated across the 5-minute period following each documented event. To ensure close temporal alignment between physiological responses and naturally occurring workplace events, HRV was assessed immediately after each event, consistent with recommendations for ambulatory psychophysiological research [54]. If additional events occurred within this time window, overlapping HRV segments were retained and treated as separate event-related observations. This approach was chosen to preserve the ecological validity of the data, as stressful situations in routine nursing work frequently occur in close temporal succession rather than as isolated incidents. In the HRV sample, 346 of 1,318 event windows (26.3%) overlapped with at least one other event window, whereas 972 event windows (73.7%) represented isolated events without overlap.

#### Metabolic equivalents

Energy expenditure was calculated using Movisens’ 2-step algorithm, which first classifies activity type based on accelerometer and barometric data and then applies regression models to estimate metabolic equivalents (METs) and energy expenditure. The computation incorporates individual parameters (age, sex, weight, height) together with movement acceleration and altitude changes. MET was included as a covariate because it is a known influencing factor of HRV [55].

### Statistical analysis

The dataset had a 2-level structure with repeated HRV measures and observer-rated stress (Level 1) nested within individuals (Level 2; see Table 1). Table 1 summarizes the analytical samples used for each model, reflecting exclusions due to missing questionnaire data, unavailable patient call systems, and invalid HRV recordings.

**Table 1:** Multilevel data structure across analytical samples

Events	Observer-rated stress sample	HRV sample
	Observations	Observations
Participants (Level 2)	85	80
Mean number of observations per participant	19.02 (*SD* = 10.81)	16.48 (*SD* = 10.91)
Range of observations per participant	1–59	1–59
Total Level 1 units	1,617 ^a^	1,318 ^b^
**Chronic stress**	**Observations**	**Observations**
Participants	76^c^	71^c^
Mean number of observations per participant	19.74 (*SD* = 10.49)	16.97 (*SD* = 10.80)
Range of observations per participant	2–59	1–59
Total Level 1 units	1,500	1,205
**Patient call system binary comparison**	**Observations**	**Observations**
Participants	85	80
Patient call system events	603 (64 nurses)	484 (59 nurses)
Non-call work events	1,617 (85 nurses)	1,318 (80 nurses)
Total Level 1 units	2,220	1,802
**Call reason**	**Observations**	**Observations**
Participants	63^d^	58^d^
Mean number of observations per participant	8.76 (*SD* = 8.59)	7.60 (*SD* = 8.27)
Range of observations per participant	1–42	1–42
Total Level 1 units	552	441

Note. ^a^ 50 of the 1,667 classified non-call work-event-level observations were excluded because observer-rated stress values were missing, leaving 1,617 observations for the observer-rated stress analyses. ^b^ Of the 1,617 event windows, 299 did not yield a valid HRV estimate and were excluded, leaving 1,318 observations for the HRV analyses. ^c^ The reduced number of participants in the chronic stress analyses reflects that some participants did not complete the questionnaire. ^d^ The reduced call-reason samples reflect both the absence of patient call systems on some wards and the restriction to events with an available and classifiable call reason

Inferential analyses were conducted using linear multilevel models (MLM) in R (Version 4.4.3; *lme4* package [56]) to account for the nested data structure. Multilevel intraclass correlation coefficients (ICC) supported this approach, with 65% of HRV variance and 47% of observer-rated stress variance attributable to between-nurse differences. Final models were refitted using restricted maximum likelihood (REML) for variance estimation. Statistical significance was evaluated at α = 0.05. Prior to model estimation, the distribution of observer-rated stress and model residuals was inspected. Although observer-rated stress showed moderate positive skewness (skew = 0.90), linear mixed-effects models assume approximate normality of the residuals rather than of the outcome variable itself. Visual inspection of residual diagnostic plots indicated only minor deviations from normality and homoscedasticity, with no pronounced floor or ceiling effects. Therefore, linear mixed-effects models were considered appropriate for the present analyses.

Sensitivity analyses using linear mixed-effects models with an AR(1) residual correlation structure, estimated with the nlme package [57], indicated only weak temporal autocorrelation (φ = 0.20). As fixed-effect estimates and statistical inferences were comparable to models without autocorrelation correction, results are reported from the more parsimonious models without AR(1).

As an additional sensitivity analysis, each multilevel model used to address the study aims was refitted including time at work and shift type as fixed-effect covariates. Time at work was operationalized as the elapsed time (in hours) since the beginning of the observation period for each participant, and shift type (early vs. late shift) was included as a categorical covariate. All other model specifications remained unchanged.

To examine which work events are stress-relevant during nurses’ everyday clinical work, 2 parallel MLMs were fitted, predicting lnRMSSD and observer-rated stress, respectively. For lnRMSSD, a baseline model including within-person centered physical activity (i.e., MET [58]) was compared with an extended model additionally including event category (clinical care burden, organizational burden, positive events) as a fixed effect, with clinical care burden as the reference. To ensure robustness, models were refitted using alternative reference categories, allowing direct estimation of all pairwise contrasts and confirming invariance of results. Across all models, random slope specifications were examined but consistently indicated overparameterization (e.g., negligible variance, high correlations, or near-unidentifiable structures). Therefore, only random-intercept-only models were retained.

For predicting observer-rated stress, we conducted a baseline intercept-only model and an extended model including event category as fixed predictor.

To examine our Sub-question 1, we extended the respective event-models for lnRMSSD and observer-rated stress by sequentially adding person-level predictors. Specifically, one model tested the influence of age and sex on HRV (lnRMSSD) and observer-rated stress, whereas a separate model examined the influence of chronic stress (i.e., PSS-10) on these variables. Chronic stress was analyzed in a separate model due to a reduced sample size resulting from missing questionnaire data. Only complete cases were included in the analysis.

To examine Sub-question 2, separate models were estimated for HRV (lnRMSSD; controlling for physical activity) and observer-rated stress, each including a binary event variable indicating whether an event was a patient call system event (1) or a non-call work event (0). Patient call system events and non-call work events represented mutually exclusive event groups.

As an exploratory subgroup analysis, we examined whether specific reasons for a call affected acute stress indicators. Call reasons were grouped into 4 broader categories, namely acute medical needs, care and mobilization support, comfort and service issues, complaints and condition, and entered as fixed effects in models predicting HRV (lnRMSSD; controlling for physical activity) and observer-rated stress. The initial categorization was performed using an LLM (ChatGPT-5), which was instructed to assign all call reasons to exactly 4 higher-level thematic clusters based on their underlying purpose, patient need, or context, generate category names, and allocate each call reason to one cluster only (for the exact prompt see Supplementary Material S2). The resulting classification was subsequently reviewed by the study team and reviewed independently by 2 domain experts. Both experts confirmed all category assignments. The final categorization was therefore accepted without modification. The mapping of individual call reasons to the 4 broader categories is provided in Supplementary Table 1. The overall effect of call reason was tested for each outcome. Tukey-adjusted pairwise contrasts between all call-reason categories were additionally estimated and reported for completeness. Acute medical needs served as the reference category.

## Results

### Demographic characteristics

The sample for observer-rated stress comprised 85 nurses (68 female, 17 male; mean age = 37.4 years, *SD* = 11.0, range: 19–62). For HRV analyses, data from 80 nurses were available, as ECG data from 5 additional participants could not be included because of technical problems with the recordings (63 female, 17 male; mean age = 37.6 years, *SD* = 11.1, range: 19–62; see Table 1 for all sample sizes per analysis). Across the 1,318 event-level HRV observations, mean RMSSD was 28.12 ms (SD = 17.02 ms; range: 3.74–178.95 ms). To provide participant-level descriptives, RMSSD was additionally averaged across events separately for each of the 80 nurses. These participant-specific mean RMSSD values ranged from 7.48 to 87.44 ms (M = 27.91 ms, SD = 13.06 ms). Due to skewed distribution, RMSSD values were natural-log-transformed (lnRMSSD) prior to analysis.

### Identification of stress-relevant events during nurses’ everyday clinical work

To identify stress-relevant work events, separate multilevel models were estimated for HRV (lnRMSSD) and observer-rated stress. Baseline models indicated moderate between-person variability (σ² = 0.17 for HRV; σ² = 1.20 for observer-rated stress), with multilevel ICCs of 0.65 and 0.47, respectively. Pairwise comparisons were based on Tukey-adjusted contrasts.

**Fig. 3:**
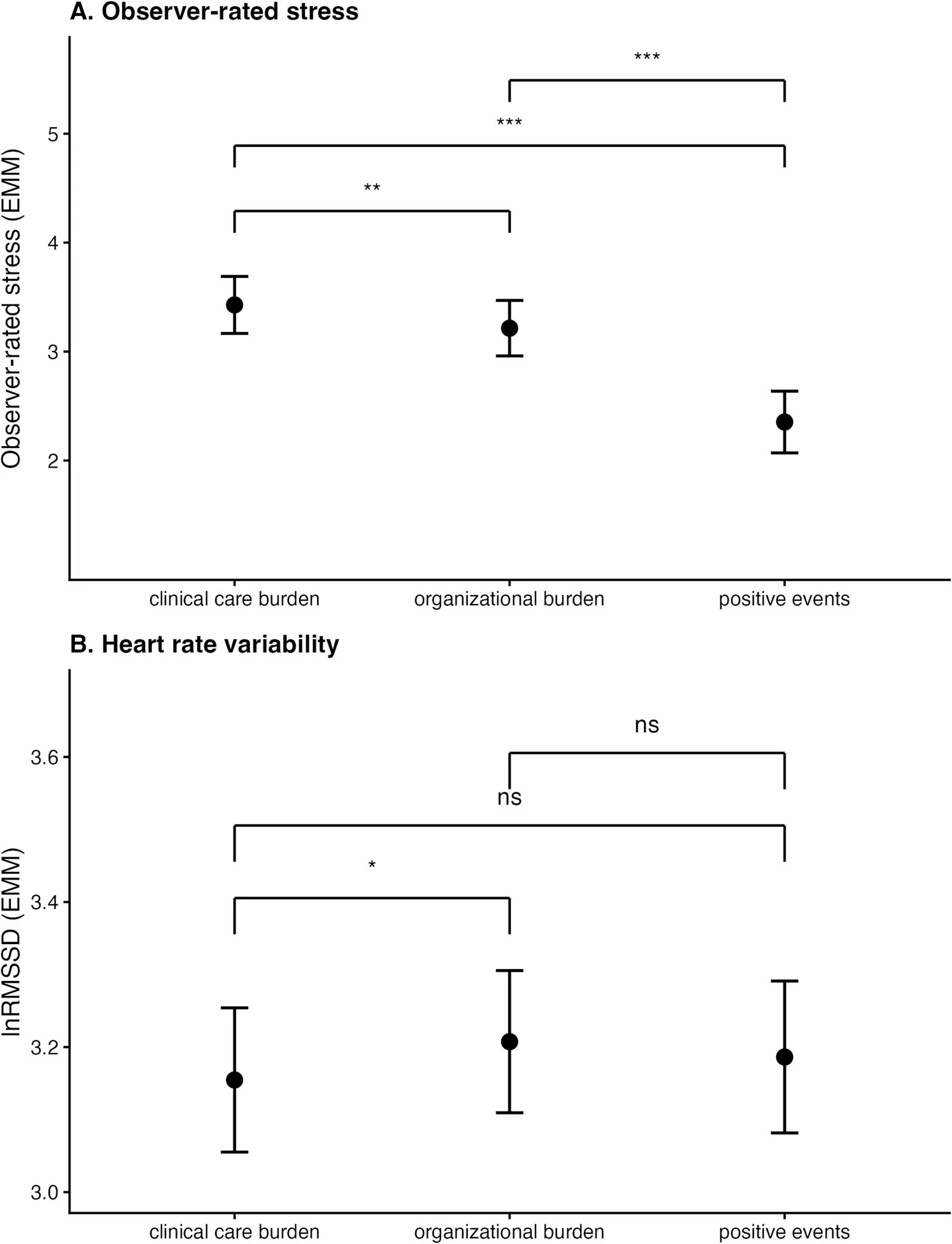
Estimated marginal means of observer-rated stress and HRV by event category. Estimated marginal means (EMMs) with 95% confidence intervals are shown for observer-rated stress and lnRMSSD across event categories. lnRMSSD estimates were adjusted for within-person centered MET. Significant pairwise differences (Tukey-adjusted) are indicated by asterisks (* *p* < .05, ** *p* < .01, *** *p* < .001; ns = not significant)

With respect to lnRMSSD, the model including event category as a predictor provided a better fit than the baseline model with MET alone, narrowly meeting the prespecified significance threshold (Δχ²(2) = 6.00, *p* = .050; unrounded *p* = .04991), with a marginal R² of 0.019. Tukey-adjusted pairwise comparisons indicated that organizational burden events were associated with significantly higher lnRMSSD than clinical care burden events (*b* = 0.053, SE = 0.022, *p* = .039). Positive events did not differ significantly from clinical care burden (*b* = 0.032, SE = 0.028, *p* = .505) or organizational burden (*b* = 0.021, SE = 0.027, *p* = .717; see Fig. 3; Table 2). With respect to observer-rated stress, the model including event category as a predictor provided a significantly better fit than the null model (Δχ²(2) = 129.93, *p* < .001), with a marginal R² of 0.046. Tukey-adjusted pairwise comparisons showed that observer-rated stress was significantly higher during clinical care burden events than during organizational burden events (*b* = 0.214, SE = 0.072, *p* = .008) and positive events (*b* = 1.075, SE = 0.094, *p* < .001). In addition, organizational burden events were associated with higher observer-rated stress levels than positive events (*b* = 0.861, SE = 0.089, *p* < .001), indicating that positive events were observer-rated as the least stressful overall (see Table 2; Fig. 3). Results were invariant to the choice of reference category.

Sensitivity analyses additionally controlling for time-at-work and shift type yielded comparable results. For observer-rated stress, the effects of event category remained virtually unchanged, whereas time-at-work was positively associated with observer-rated stress (b = 0.09, SE = 0.01, *p* < .001) and shift type showed no significant association (*p* = .360). Similarly, for lnRMSSD, the event-category effects remained stable after adjustment, while time-at-work was associated with lower lnRMSSD (b = − 0.01, SE = 0.004, *p* = .016) and shift type was not significantly associated with lnRMSSD (*p* = .692). These findings indicate that the observed event-category effects were robust to temporal and shift-related influences.

In sum, clinical care burden was consistently associated with the highest observer-rated stress levels and positive events with the lowest. Observer-rated stress differentiated all 3 event categories, whereas lnRMSSD distinguished only clinical care burden from organizational burden, with positive events not significantly different from either. At the same time, the marginal R² values were small, suggesting that nurses’ psychophysiological responses are influenced by a range of additional situational and individual factors beyond the event classifications examined in the present study.

**Table 2:** Tukey-adjusted pairwise contrasts by event category

Outcome	Contrast	b	SE	t (df)	*p*	CI
lnRMSSD	clinical care burden – organizational burden	-0.053	0.022	-2.45 (1269)	**0.039**	[-0.103, -0.002]
lnRMSSD	clinical care burden – positive events	-0.032	0.028	-1.12 (1259)	0.505	[-0.098, 0.035]
lnRMSSD	organizational burden – positive events	0.021	0.027	0.78 (1261)	0.717	[-0.043, 0.085]
Observer-rated stress	clinical care burden – organizational burden	0.214	0.072	2.97 (1587)	**0.008**	[0.045, 0.382]
Observer-rated stress	clinical care burden – positive events	1.075	0.094	11.38 (1562)	**< 0.001**	[0.853, 1.297]
Observer-rated stress	organizational burden – positive events	0.861	0.089	9.68 (1561)	**< 0.001**	[0.653, 1.070]

Note. Estimates are derived from the final multilevel models. lnRMSSD contrasts are adjusted for within-person centered MET

### Influence of individual characteristics on event-related stress

**Fig. 4:**
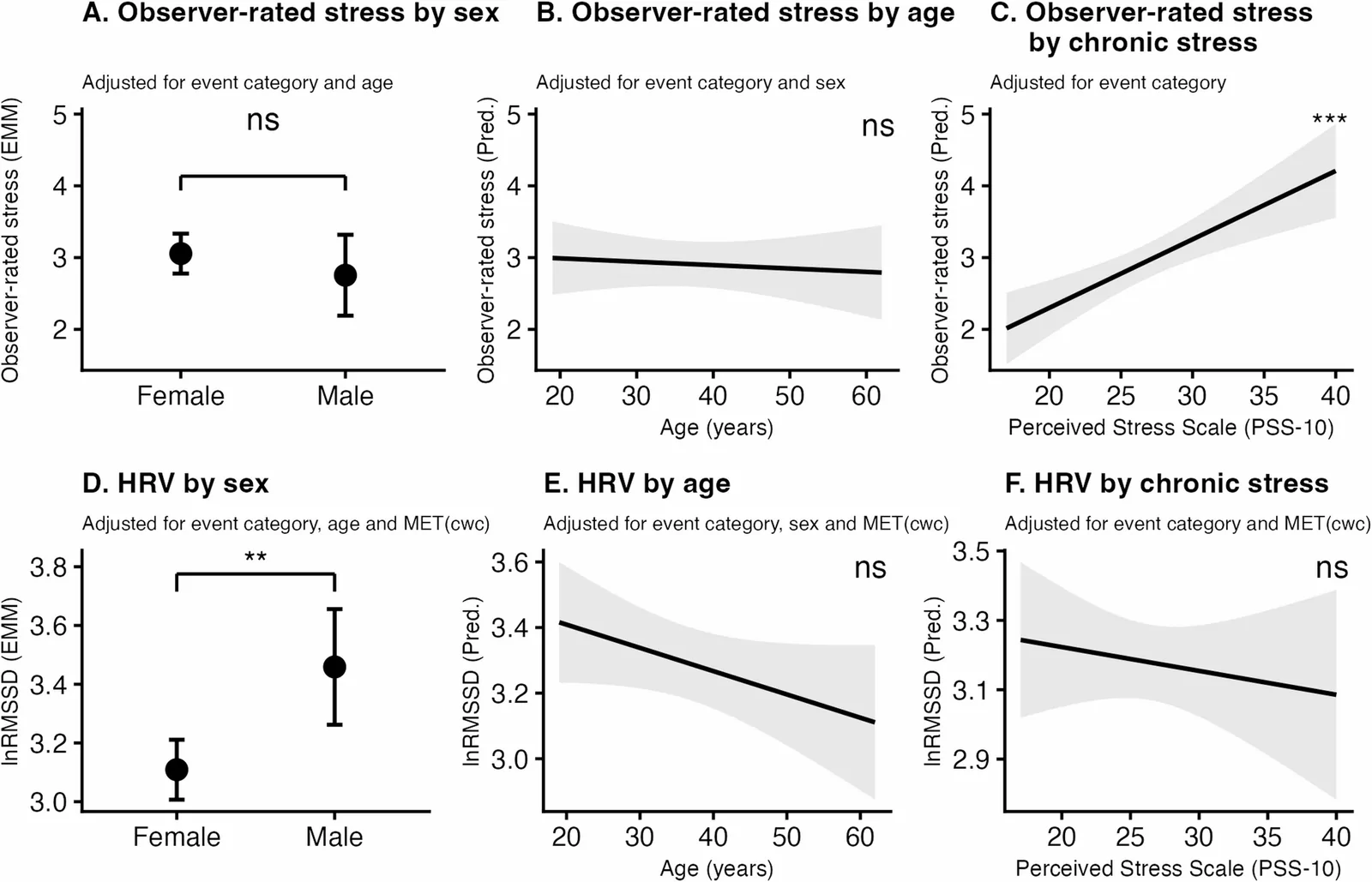
Effects of individual characteristics on observer-rated stress and HRV. Estimated marginal means (EMMs) with 95% confidence intervals are shown for sex. For the continuous predictors age and chronic stress (PSS-10), model-predicted values (Pred.) with 95% confidence intervals are displayed. lnRMSSD models were additionally adjusted for within-person centered MET (MET[cwc]; cwc = centering within cluster). Significant effects are indicated by asterisks (* *p* < .05, ** *p* < .01, *** *p* < .001; ns = not significant)

Linear mixed models, corresponding to the previously described models for lnRMSSD and observer-rated stress, were extended to examine the influence of individual characteristics (see Table 3; Fig. 4).

For observer-rated stress, including age and sex simultaneously did not significantly improve model fit (Δχ²(2) = 1.04, *p* = .594). As illustrated in Fig. 4A and B, neither sex nor age showed a significant association with observer-rated stress. In contrast, including chronic stress (PSS-10) significantly improved model fit compared to the event-only model (Δχ²(1) = 16.15, *p* < .001). Higher chronic stress scores were associated with higher observer-rated stress (*b* = 0.10, SE = 0.02, 95% CI [0.05, 0.14], *p* < .001; see Fig. 4C).

For lnRMSSD, extending the model by including age and sex simultaneously significantly improved model fit (Δχ²(2) = 13.38, *p* = .001). Within this model, sex was significantly associated with lnRMSSD (*b* = 0.35, SE = 0.11, 95% CI [0.136, 0.564], *p* = .002; see Fig. 4D), with male nurses showing generally higher lnRMSSD values independent of event category. Given the small number of male participants, this finding should be considered exploratory. Age showed a non-significant negative estimate (*b* = -0.007, SE = 0.004, 95% CI [-0.015, 0.001], *p* = .085; see Fig. 4E). In contrast, adding chronic stress (PSS-10) did not significantly improve model fit for lnRMSSD (Δχ²(1) = 0.45, *p* = .504; see Fig. 4F), indicating no evidence for an association between chronic stress and lnRMSSD in the present sample.

Sensitivity analyses additionally controlling for time-at-work and shift type did not materially change the observed associations between the individual characteristics and either outcome.

Importantly, event category effects remained robust after controlling for age, sex, and chronic stress, indicating that the observed associations were not explained by individual differences.

**Table 3:** Model comparisons for individual characteristics

Outcome	Comparison	Model comparison
Observer-rated stress	Add age and sex	Δχ²(2) = 1.04, *p* = .594
Observer-rated stress	Add chronic stress (PSS-10)	Δχ²(1) = 16.15, ***p*< .001**
lnRMSSD	Add age and sex	Δχ²(2) = 13.38, ***p*= .001**
lnRMSSD	Add chronic stress (PSS-10)	Δχ²(1) = 0.45, *p* = .504

### Influence of patient call system events on stress indicators

**Fig. 5:**
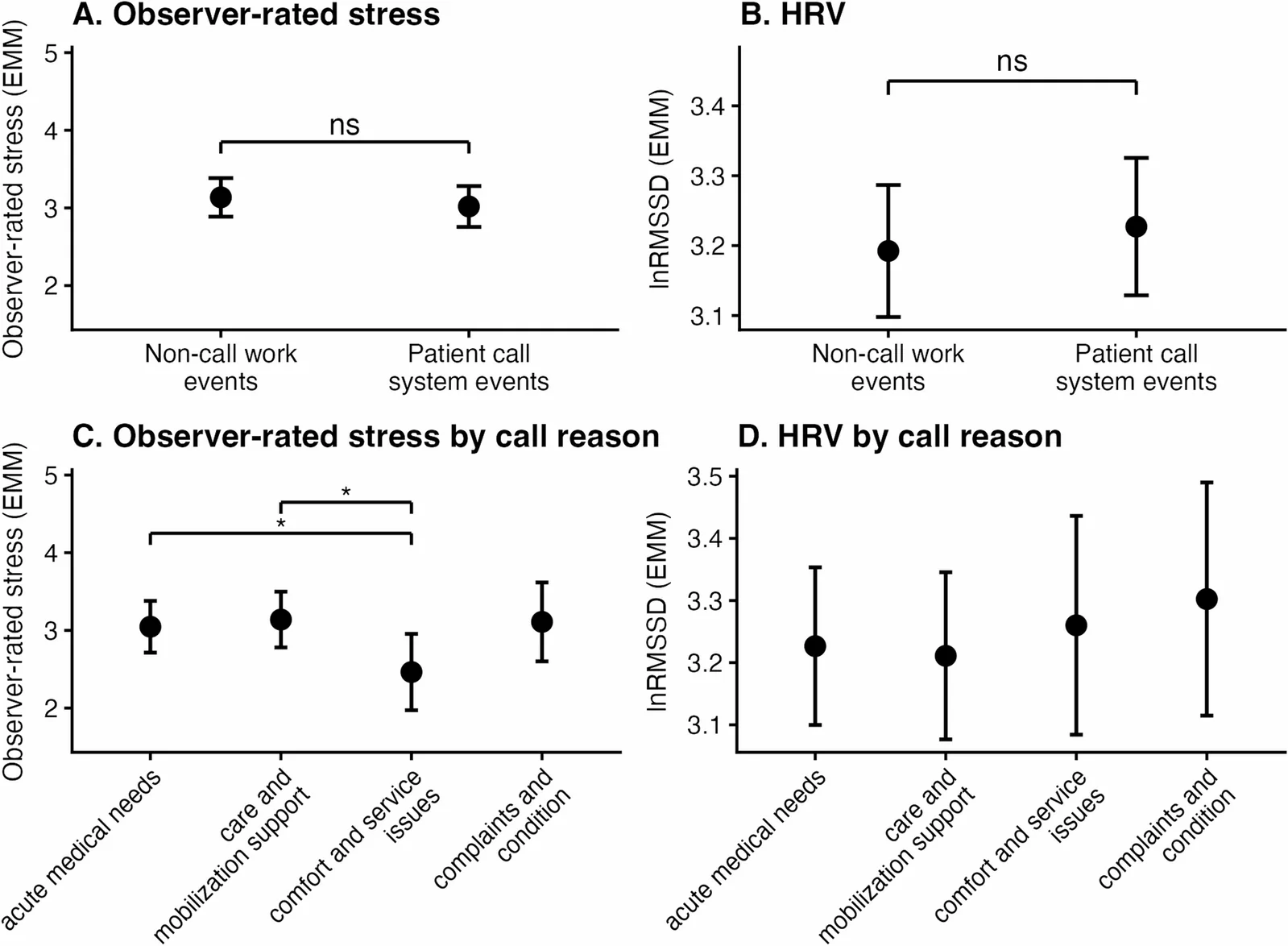
Estimated marginal means of observer-rated stress and HRV for patient call system events and call reasons. Estimated marginal means (EMMs) with 95% confidence intervals are shown for (**A**) observer-rated stress and (**B**) lnRMSSD comparing patient call system events with non-call work events, and for (**C**) observer-rated stress and (**D**) lnRMSSD across different call reason categories (acute medical needs, care and mobilization support, comfort and service issues, and complaints and condition). lnRMSSD estimates were adjusted for within-person centered MET. Asterisks indicate statistical significance (* *p* < .05, ** *p* < .01, *** *p* < .001, ns = not significant)

The binary observer-rated stress model included 2,220 event-level observations from 85 nurses, comprising 603 patient call system events for which the observed nurse was responsible (64 nurses) and 1,617 non-call work events (85 nurses). The corresponding HRV model included 1,802 event-level observations from 80 nurses, comprising 484 patient call system events for which the observed nurse was responsible (59 nurses) and 1,318 non-call work events (80 nurses). Patient call system events and non-call work events thus represented mutually exclusive event groups. Patient call system events as a binary predictor did not significantly differ from non-call work events in observer-rated stress (Δχ²(1) = 3.24, *p* = .072, *b* = − 0.118, SE = 0.065, 95% CI [− 0.246, 0.010]) or lnRMSSD (Δχ²(1) = 2.96, *p* = .085, *b* = 0.035, SE = 0.020, 95% CI [− 0.005, 0.075]; adjusted for MET) (see Fig. 5A and B). Descriptively, observer-rated stress was comparable between patient call system events (*n* = 603 observations, 64 nurses; raw mean = 3.14, SD = 1.64, median = 3, range = 1–9) and non-call work events (*n* = 1,617 observations, 85 nurses; raw mean = 3.12, SD = 1.59, median = 3, range = 1–10). Likewise, mean lnRMSSD was comparable for patient call system events (*n* = 484 observations, 59 nurses; raw mean = 3.20, SD = 0.67, median = 3.26, range = 1.05–5.17) and non-call work events (*n* = 1,318 observations, 80 nurses; raw mean = 3.19, SD = 0.54, median = 3.24, range = 1.32–5.19).

### Influence of specific call reasons on stress indicators

As an exploratory subgroup analysis, we examined whether the specific reason for a patient call influenced nurses’ stress levels by estimating separate multilevel models for lnRMSSD and observer-rated stress (see Fig. 5C and D). The model predicting lnRMSSD controlling for physical activity including call reason as a fixed effect did not significantly improve model fit compared with the baseline model including within-person centered MET (Δχ²(3) = 1.34, *p* = .720), with a marginal R² of 0.003, suggesting no evidence for systematic differences in lnRMSSD across call-reason categories in the present sample (see Fig. 5D and Supplementary Table 2).

In contrast, the model predicting observer-rated stress including call reason as a fixed effect showed a significantly better fit compared to the intercept-only model (Δχ²(3) = 9.44, *p* = .024; *n* = 552, 63 nurses), with a marginal R² of 0.009. Post-hoc Tukey-adjusted comparisons revealed that comfort and service issues were associated with significantly lower observer-rated stress compared to both acute medical needs (*b* = 0.58, SE = 0.22, *t*(523) = 2.68, *p* = .038) and care and mobilization support (*b* = 0.68, SE = 0.22, *t*(519) = 3.03, *p* = .014; see Fig. 5C and Supplementary Table 3). No other pairwise comparisons between call reasons were significant.

Taken together, this exploratory analysis suggests that patient call system events did not significantly differ from non-call work events in either observer-rated stress or HRV, whereas the specific call reason was associated with observer-rated stress only, with calls related to comfort and service issues rated less stressful than acute medical or care and mobilization support calls. No corresponding differences were observed for HRV (see Fig. 5D).

Comparable sensitivity analyses were conducted for the patient call system models. The inclusion of time-at-work and shift type did not materially alter the interpretation of these findings.

## Discussion

This study provides preliminary evidence that an EMA-based psychophysiological field assessment is both feasible during nurses’ regular shifts and sensitive enough to identify stress-relevant events in everyday clinical work. Specifically, clinical care burden consistently emerged as the event category associated with the highest observer-rated stress, event effects remained robust after controlling for individual characteristics, and patient call system events did not significantly differ from non-call work events in observer-rated stress or HRV. However, observer-rated stress varied by call reason.

With regard to the main research question, namely, which events during nurses’ everyday clinical work are associated with changes in psychophysiological stress indicators, the present findings provide evidence that acute stress varies systematically depending on the type of event. More precisely, the central finding of this study is that clinical care burden events, including direct patient care activities, were associated with higher observer-rated stress than both organizational burden and positive events, and with lower HRV than organizational burden events (i.e., documentation tasks, interruption by phone). Thus, with the present event categorization, clinical care burden was associated with comparatively higher observer-rated stress and lower HRV than organizational burden and with higher observer-rated stress than both other event categories. Positive events were rated as least stressful compared to clinical care burden and organizational burden by observers, but did not differ significantly from clinical care burden events in terms of HRV. This prominent role of clinical care burden aligns with existing research showing that high job demands in nursing, particularly workload and emotional demands arising from patient care, are consistently associated with stress and its adverse consequences [4, 59, 60]. While these findings are largely based on retrospective and aggregated assessments, the present study extends this evidence by demonstrating that these associations hold at the level of concrete, real-time events, captured both physiologically and behaviorally during nurses’ regular shifts.

One possible explanation is that clinical care burden events more directly engage emotional and cognitive resources, for example when nurses must respond to unpredictable patient behavior, acute deterioration, or rapid decision-making under uncertainty, whereas organizational demands, although potentially frustrating, may be experienced as more routine and controllable and are therefore less likely to be associated with elevated stress levels. Within the JD-R framework, such situations may represent qualitatively different forms of job demands than organizational tasks, as they are characterized by greater emotional involvement, urgency, and lower perceived controllability. From a stress-theoretical perspective, these characteristics may increase the perceived urgency and reduce the controllability of the situation, both of which are likely to influence cognitive appraisal and contribute to stronger stress responses. Notably, organizational demands such as documentation constitute a substantial proportion of nurses’ workload and have been identified as key targets for improving workflow efficiency [61]. In addition, documentation activities, particularly organizational documentation, have been associated with higher perceived workload among nurses [62]. However, as these findings are typically based on aggregated self-reports, they capture general perceptions of workload rather than stress responses tied to specific situations, making it more difficult to attribute stress to distinct event types. In contrast, real-time assessment captures acute stress as it unfolds in response to concrete events, which may lead to clinical care situations standing out more clearly as primary stressors, a distinction that retrospective methods may not fully capture. If replicated in larger and more diverse samples, these findings may highlight the added value of translating abstract job demands into concrete, event-based targets for improving nurses’ working conditions and guiding interventions. Sensitivity analyses further indicated that although observer-rated stress increased and HRV decreased over the course of the work shift, adjusting for time-at-work and shift type did not alter the observed associations between event categories and stress outcomes. This suggests that the identified event-related differences cannot be explained solely by temporal changes during the workday.

At the same time, differences between the two stress indicators should be considered. While both indicators converged on clinical care burden as the most stress-relevant category, only observer-rated stress showed consistent differentiation across event categories, whereas HRV showed more limited sensitivity. Importantly, the overall HRV effect was small (marginal R² = 0.019), the model comparison only narrowly met the prespecified significance threshold, and only one pairwise contrast reached statistical significance. Therefore, the HRV findings should be interpreted cautiously and considered preliminary. Given the small effect size and the number of outcomes and pairwise comparisons examined, these findings should be considered exploratory until replicated in independent samples. One possible explanation is that stress-related changes in physiological arousal, as indexed by HRV, may only become detectable beyond a certain threshold of affective arousal [63], as supported by prior research showing stronger associations for highly arousing negative stimuli than for positive or mixed-valence experiences [64, 65]. In addition, positive events in clinical practice may often co-occur with ongoing demands, attenuating potential recovery effects in HRV [66]. More generally, this pattern is consistent with a longstanding body of research indicating that subjective and physiological affect indicators do not necessarily converge [67, 68]. Finally, as HRV reflects cardiac autonomic regulation rather than stress per se, it may capture a range of physiological states and may therefore be less specific to subtle or context-dependent stress experiences in real-world settings [69].

With regard to Sub-question 1, namely how individual characteristics influence nurses’ event-related stress indicators, the present findings indicate that these characteristics were differentially associated with the two stress indicators. An exploratory association between sex and HRV was observed, with male nurses in the present sample exhibiting higher HRV compared to female nurses in the 5-minute window following event-triggered episodes, independent of the nature of the triggering event. Given the small number of male participants and the imbalanced sex distribution, this finding should be interpreted with considerable caution and not be taken as evidence of a generalizable sex difference in HRV among nurses. This finding is difficult to directly compare with existing literature, as evidence on sex differences in HRV is highly inconsistent depending on the time window, physiological state, and specific measure used. Notably, a previous meta-analysis found no consistent sex differences in RMSSD, whereas women showed greater relative cardiac autonomic regulation as indexed by higher high-frequency (HF) power in short-term recordings [70].

Chronic stress, in contrast, was significantly associated with higher observer-rated stress across situations, independent of event category. This is consistent with evidence suggesting that chronic or background stress may modulate acute stress responses [71, 72]. The finding that chronic stress was associated with higher observer-rated, but not physiological stress may reflect that observer ratings are more sensitive to visible and interaction-related manifestations of stress in nurses’ everyday work. In this context, empathic or embodied stress may be relevant, as nurses may resonate with the distress of patients and colleagues in ways that are behaviorally salient without necessarily being mirrored in HRV [73]. Overall, the findings highlight the need to account for individual differences in everyday clinical stress.

With respect to Sub-question 2, namely whether patient call system events differ from non-call work events in their impact on nurses’ stress indicators and whether the specific reason for the call influences stress indicators, patient call system events did not differ significantly from all non-call work events. When differentiating between call reasons, calls related to comfort and service issues were rated as less stressful than those related to acute medical needs or care and mobilization support by the observer. Importantly, these differences were evident only in observer-rated stress and not in HRV. However, these findings should be interpreted cautiously, as the call-reason analyses were based on a reduced sample size compared to the primary analyses.

Although no statistically significant differences were observed between patient call system events and non-call work events, this finding may appear counterintuitive given the face validity of alarm-related interruptions as potentially stressful and disruptive in clinical practice. However, evidence from a recent scoping review on alarm fatigue conducted by our group suggests that stress and desensitization primarily arise in response to frequent and repeated alarms rather than isolated signals [46]. From this perspective, individual patient call system events may be perceived as routine and therefore not sufficiently salient to elicit stress responses, whereas their cumulative burden over time may be more relevant. As the present study focused on discrete events, such cumulative effects could not be fully captured. Moreover, patient call system events may be associated with a certain degree of control, as nurses can often decide when and how to respond. According to the Job Demand-Control model, high demands are less likely to result in stress when accompanied by higher levels of control or autonomy [74]. From this perspective, individual call events may not only be less stressful but may also represent manageable and predictable aspects of nurses’ workflow, which may contribute to lower perceived stress at the event level and may help explain why patient call system events were not associated with significantly higher acute stress in the present study. A similar interpretation may also be applied when differentiating between call reasons. Calls related to acute medical needs or care and mobilization support likely involve higher urgency, responsibility, and complexity, whereas calls related to comfort and service issues are more predictable and less demanding, resulting in lower perceived stress.

### Limitations

Several limitations should be considered. First, events were grouped into broader categories to ensure sufficient statistical power and interpretability, which may have obscured variability between specific situations and attenuated potential effects. Future research may benefit from more fine-grained or data-driven event classifications to better capture the heterogeneity of clinical work. Second, psychological stress was assessed exclusively via observer ratings, without incorporating nurses’ subjective self-reports. Although this approach reduced disruption during work, it remains unclear to what extent observer-rated stress reflects nurses’ internal experiences versus externally observable stress expressions. Moreover, observer ratings do not directly capture the subjective cognitive appraisal processes emphasized in the Transactional Stress Model [5]. Given the ICC(3,1) of 0.449, some variability in observer-rated stress may have reflected differences between observers. This measurement error may have reduced the precision of the observer-rated stress measure and attenuated the estimated associations. Future research should therefore validate observer-based assessments against nurses’ self-reported stress levels to better establish their convergent validity. This could be achieved, for example, by collecting brief momentary self-reports at a small number of predefined time points or following selected events, thereby minimizing disruption to routine clinical work while enabling direct comparison between observer- and self-rated stress. Third, the event-based design focused on discrete situations and may not fully capture cumulative or frequency-related effects of stressors, such as those described in the context of alarm fatigue. Repeated exposure to certain demands may have more substantial effects over time than can be detected at the level of individual events. Fourth, the observational nature of the study precludes causal inferences. Although the event-based design allows for the examination of associations between work events and concurrent stress indicators in real-world settings, it does not permit conclusions about the directionality or causality of these relationships, particularly given the dynamic and context-dependent nature of clinical work. Furthermore, the comparison group in the analyses using patient call system events as a binary predictor included work events from wards both with and without a patient call system. Consequently, differences between patient call system events and non-call work events may partly reflect differences in ward characteristics or work organization associated with patient call system availability. Fifth, the analyses of specific call reasons were exploratory and based on a substantially smaller sample than the primary analyses, which may have limited statistical power and the precision of the estimated effects.

In addition, participants were recruited from a single German university hospital. Working conditions, staffing structures, and organizational processes may differ across healthcare systems, hospital types, and countries. Consequently, the generalizability of the findings to other clinical settings should be interpreted with caution and requires replication in more diverse samples.

### Implications

Methodologically, the present findings demonstrate the feasibility of combining continuous physiological monitoring with event-based assessment to capture event-related changes in observer-rated and physiological stress indicators in real time during nurses’ regular work shifts. This extends existing approaches that rely on retrospective or aggregated measures and highlights the potential of EMA to identify stress-relevant situations with high temporal and contextual specificity. At the same time, although fewer effects were detected using physiological measures compared to observer-rated stress, HRV still appeared to capture meaningful variations in cardiac autonomic regulation. This suggests that HRV-based monitoring may have potential for identifying salient or activating situations over extended periods. Importantly, such approaches can be implemented without disrupting nurses’ workflow, and ongoing advances in wearable sensor technology are likely to further enhance their feasibility, scalability, and cost-effectiveness in real-world clinical settings. Future research should further refine event-based methodologies by developing more fine-grained and context-sensitive event classifications, as the current categorization still represents a relatively abstract level that may limit direct translation into highly specific workplace interventions.

From a practical perspective, the results suggest that efforts to improve nurses’ working conditions may benefit from focusing on specific high-burden clinical situations, particularly those related to direct patient care, rather than addressing job demands only at a general level, if replicated across different healthcare settings. Interventions targeting emotionally and cognitively demanding patient-care situations may be especially relevant for reducing acute stress in everyday clinical practice. Examples may include training in de-escalation and communication strategies for challenging interactions with patients or relatives, structured escalation procedures during acute patient deterioration, team-based backup systems during periods of high workload, or technology-supported interventions that provide real-time stress management support [75, 76]. For example, wearable sensor systems may enable just-in-time adaptive interventions by detecting stress-related physiological changes and delivering brief evidence-based self-regulation strategies such as slow-paced breathing exercises [77–79].

Beyond this, the study highlights the potential of integrating event-based stress assessment into increasingly digitalized healthcare environments. Such approaches may help identify stress-relevant clinical processes that could benefit from digital support in comparable healthcare settings. In particular, they may allow for assessing whether digital interventions reduce stress-relevant events or inadvertently introduce new sources of stress in routine care.

## Conclusion

This study suggests that acute stress in nursing varies systematically across everyday work events. Within the event categorization used in this study, clinical care burden emerged as the most consistent stress-related category. By combining event-based assessment with physiological monitoring, the findings highlight a promising foundation for developing scalable approaches to identify stress-relevant situations in nurses’ real-world clinical practice.

## Supplementary Information

Below is the link to the electronic supplementary material.

Supplementary Material 1

Supplementary Material 2

## Data Availability

The datasets generated and/or analyzed during the current study are available from the corresponding author on reasonable request. Due to the sensitive nature of the data (including physiological and observational data collected in a clinical setting), data are not publicly available but can be shared in anonymized form upon request. The Code used to analyze the data can be found here: https://osf.io/uxsc2/overview? view_only=4943201a7b0c4fa1854be74f128f230a.

## Abbreviations

CI: Confidence interval
cwc: centering within cluster
ECG: Electrocardiogram
EMA: Ecological momentary assessment
HRV: Heart rate variability
ICC: Intraclass correlation coefficient
ICU: Intensive care unit
JD-R: Job demands-resources
LLM: Large language model
lnRMSSD: Natural logarithm of the root mean square of successive differences between successive RR intervals
MET: Metabolic equivalent of task
MLM: Multilevel model
NN: Normal-to-normal
PSS-10: Perceived stress scale (10 items)
REML: Restricted maximum likelihood
RMSSD: Root mean square of successive differences between successive RR intervals
VAS: Visual analogue scale
