# Supplementary material for: Identifying stress-relevant events in everyday nursing practice using psychophysiological outcomes - an observational multimethod study
Source: BMC Nurs. 2026 Sep 29;25:899. doi: 10.1186/s12912-026-05434-w (PMC13625228; doi:10.1186/s12912-026-05434-w)
Supplement: Supplementary file 1 [file 12912_2026_5434_MOESM1_ESM.docx]

# Supplementary Material

**S1. Prompt used for AI-assisted clustering of event categories (ChatGPT-5):**

“Assign the following events to exactly three higher-level thematic clusters. Create the clusters based on the underlying cause or context of each event rather than on their frequency or severity. Give each cluster a concise and meaningful name, and assign every event to exactly one cluster.”

**S2. Prompt used for AI-assisted clustering of call reasons (ChatGPT-5):**

“Assign the following call bell reasons to exactly four higher-level thematic clusters. Create the clusters based on the underlying purpose, patient need, or context of each call bell reason rather than on their frequency or urgency. Give each cluster a concise and meaningful name, and assign every call bell reason to exactly one cluster.”
