# Supplementary material for: Identifying stress-relevant events in everyday nursing practice using psychophysiological outcomes - an observational multimethod study
Source: BMC Nurs. 2026 Sep 29;25:899. doi: 10.1186/s12912-026-05434-w (PMC13625228; doi:10.1186/s12912-026-05434-w)
Supplement: Supplementary file 2 [file 12912_2026_5434_MOESM2_ESM.docx]

# Supplementary Tables

**Supplementary Table 1. Classification of observed work events and call reasons**

The table presents the 31 non-call work-event types used for the primary event classification and the 15 classifiable patient call reasons used in the call-reason analysis, together with their assigned categories and observed frequencies.

| **Event description** | **Event category** | **Frequency** |
| --- | --- | --- |
| *Clinical care burden events* |  | *552* |
| Additional call bell during patient care | clinical care burden | 138 |
| Problems with patient call bell | clinical care burden | 2 |
| Work in isolation room | clinical care burden | 70 |
| Disoriented patient requires attention | clinical care burden | 14 |
| Patient fall | clinical care burden | 0 |
| Patient circulation unstable | clinical care burden | 20 |
| Resuscitation | clinical care burden | 0 |
| Resuscitation on ward | clinical care burden | 0 |
| Transfer patient from operating room/recovery room | clinical care burden | 20 |
| Demanding or impatient relatives | clinical care burden | 26 |
| No physician available | clinical care burden | 7 |
| Demanding or difficult patient | clinical care burden | 216 |
| Monitoring alarm | clinical care burden | 39 |
| *Organizational burden events* |  | *875* |
| Nurse expresses work overload | organizational burden | 22 |
| Sudden tasks exceeding staffing capacity | organizational burden | 44 |
| Admission or discharge of a patient | organizational burden | 38 |
| Problems with access (e.g., IV line) | organizational burden | 27 |
| Interruption by phone | organizational burden | 427 |
| Unplanned discharge | organizational burden | 1 |
| Missing equipment | organizational burden | 88 |
| Uncertainties in care process | organizational burden | 94 |
| Change in therapy procedure | organizational burden | 31 |
| New order for blood sampling or examination | organizational burden | 27 |
| Nurse reports extensive documentation workload | organizational burden | 15 |
| Searching for help or support | organizational burden | 61 |
| *Positive events* |  | *240* |
| Patient or relative expresses verbal gratitude | positive events | 61 |
| Patient or relative gives gifts or money | positive events | 16 |
| Spontaneous support from colleagues | positive events | 94 |
| Recognition from colleagues or supervisors | positive events | 21 |
| Successful acute therapy | positive events | 2 |
| Other positive event | positive events | 46 |
| **Patient call reason** | **Call reason category** | **Frequency** |
| *Acute medical needs* |  | *356* |
| Device alarm | acute medical needs | 101 |
| Infusion empty | acute medical needs | 205 |
| False emergency alarm | acute medical needs | 13 |
| Disconnecting patient from infusion/monitoring | acute medical needs | 37 |
| *Care and mobilization support* |  | *134* |
| Toileting | care and mobilization support | 97 |
| Repositioning/assistance with mobilization | care and mobilization support | 24 |
| Assistance with personal hygiene | care and mobilization support | 13 |
| *Comfort and service issues* |  | *35* |
| Food/drink request | comfort and service issues | 17 |
| Light operation | comfort and service issues | 6 |
| Picking up objects | comfort and service issues | 3 |
| Window/blinds operation | comfort and service issues | 8 |
| Call related to roommate | comfort and service issues | 1 |
| *Complaints and condition* |  | *30* |
| Pain | complaints and condition | 13 |
| Discomfort | complaints and condition | 12 |
| Inquiry about medical rounds | complaints and condition | 5 |

**Note.** Frequencies for the 3 primary event categories are based on all observed non-call work events (n = 1,667); patient call system events were excluded from these frequencies and analyzed separately. Of the 1,667 non-call work events, 50 observations without an observer-rated stress value were excluded from the observer-rated stress analyses, resulting in an analytical sample of 1,617 observations. Of these 1,617 event windows, 299 did not yield a valid HRV estimate and were excluded from the HRV analyses, resulting in 1,318 event-level HRV observations. Frequencies for patient call reasons are based on all patient call system events with an available and classifiable call reason (n = 555), irrespective of outcome-specific data availability. The corresponding analytical samples comprised 552 observations for observer-rated stress and 441 observations for HRV.

**Supplementary Table 2. Tukey-adjusted pairwise contrasts for call reasons and lnRMSSD**

| Contrast | b | SE | t (df) | *p* | CI |
| --- | --- | --- | --- | --- | --- |
| acute medical needs - care and mobilization support | 0.02 | 0.05 | 0.34 (420.18) | .987 | [-0.10, 0.14] |
| acute medical needs - comfort and service issues | -0.03 | 0.07 | -0.45 (410.86) | .970 | [-0.23, 0.16] |
| acute medical needs - complaints and condition | -0.08 | 0.08 | -0.91 (416.53) | .802 | [-0.29, 0.14] |
| care and mobilization support - comfort and service issues | -0.05 | 0.08 | -0.64 (407.77) | .919 | [-0.25, 0.15] |
| care and mobilization support - complaints and condition | -0.09 | 0.09 | -1.05 (416.78) | .718 | [-0.32, 0.13] |
| comfort and service issues - complaints and condition | -0.04 | 0.10 | -0.41 (410.92) | .977 | [-0.31, 0.22] |

**Supplementary Table 3. Tukey-adjusted pairwise contrasts for call reasons and observer-rated stress**

| Contrast | b | SE | t (df) | *p* | CI |
| --- | --- | --- | --- | --- | --- |
| acute medical needs - care and mobilization support | -0.09 | 0.13 | -0.70 (532.83) | .896 | [-0.43, 0.25] |
| acute medical needs - comfort and service issues | 0.58 | 0.22 | 2.68 (522.69) | .038 | [0.02, 1.14] |
| acute medical needs - complaints and condition | -0.06 | 0.23 | -0.27 (525.35) | .993 | [-0.65, 0.53] |
| care and mobilization support - comfort and service issues | 0.68 | 0.22 | 3.03 (518.73) | .014 | [0.10, 1.25] |
| care and mobilization support - complaints and condition | 0.03 | 0.24 | 0.13 (525.07) | .999 | [-0.58, 0.64] |
| comfort and service issues - complaints and condition | -0.65 | 0.29 | -2.21 (519.48) | .121 | [-1.40, 0.11] |
